# Ontology-based feature engineering in machine learning workflows for heterogeneous epilepsy patient records

**DOI:** 10.1038/s41598-022-23101-3

**Published:** 2022-11-12

**Authors:** Satya S. Sahoo, Katja Kobow, Jianzhe Zhang, Jeffrey Buchhalter, Mojtaba Dayyani, Dipak P. Upadhyaya, Katrina Prantzalos, Meenakshi Bhattacharjee, Ingmar Blumcke, Samuel Wiebe, Samden D. Lhatoo

**Affiliations:** 1 Department of Population and Quantitative Health Sciences, Case Western Reserve University, Cleveland, OH USA; 2 Institute of Neuropathology, Erlangen, Germany; 3 Department of Neurology, University of Texas Health Sciences Center, Texas, USA; 4 Department of Pediatrics, University of Calgary School of Medicine, Calgary, Canada

**Keywords:** Epilepsy, Information technology, Scientific data, Software

## Abstract

Biomedical ontologies are widely used to harmonize heterogeneous data and integrate large volumes of clinical data from multiple sources. This study analyzed the utility of ontologies beyond their traditional roles, that is, in addressing a challenging and currently underserved field of feature engineering in machine learning workflows. Machine learning workflows are being increasingly used to analyze medical records with heterogeneous phenotypic, genotypic, and related medical terms to improve patient care. We performed a retrospective study using neuropathology reports from the German Neuropathology Reference Center for Epilepsy Surgery at Erlangen, Germany. This cohort included 312 patients who underwent epilepsy surgery and were labeled with one or more diagnoses, including dual pathology, hippocampal sclerosis, malformation of cortical dysplasia, tumor, encephalitis, and gliosis. We modeled the diagnosis terms together with their microscopy, immunohistochemistry, anatomy, etiologies, and imaging findings using the description logic-based Web Ontology Language (OWL) in the Epilepsy and Seizure Ontology (EpSO). Three tree-based machine learning models were used to classify the neuropathology reports into one or more diagnosis classes with and without ontology-based feature engineering. We used five-fold cross validation to avoid overfitting with a fixed number of repetitions while leaving out one subset of data for testing, and we used recall, balanced accuracy, and hamming loss as performance metrics for the multi-label classification task. The epilepsy ontology-based feature engineering approach improved the performance of all the three learning models with an improvement of 35.7%, 54.5%, and 33.3% in logistics regression, random forest, and gradient tree boosting models respectively. The run time performance of all three models improved significantly with ontology-based feature engineering with gradient tree boosting model showing a 93.8% reduction in the time required for training and testing of the model. Although, all three models showed an overall improved performance across the three-performance metrics using ontology-based feature engineering, the rate of improvement was not consistent across all input features. To analyze this variation in performance, we computed feature importance scores and found that microscopy had the highest importance score across the three models, followed by imaging, immunohistochemistry, and anatomy in a decreasing order of importance scores. This study showed that ontologies have an important role in feature engineering to make heterogeneous clinical data accessible to machine learning models and also improve the performance of machine learning models in multilabel multiclass classification tasks.

## Introduction

The growing role of artificial intelligence (AI) and in particular machine learning algorithms in biomedical research domains have highlighted both opportunities as well as challenges in effectively using large-scale biomedical datasets^1–3^. The availability of large volumes of clinical data together with a variety of machine learning models represent key opportunities; however, data heterogeneity and the availability of limited data harmonization techniques present critical bottlenecks. These challenges were recently highlighted by the US National Institutes of Health (NIH) Bridge2AI initiative^4^. The Bridge2AI initiative focuses on the critical need to make biomedical data “Artificial Intelligence/Machine Learning (AI/ML) ready” using ontologies and terminologies as a core component for “AI/ML readiness”.

The challenge of data heterogeneity is particularly acute in epilepsy neurological disorder due to its disparate clinical phenotype, etiologies, mechanism of seizures, genetics, and related medical conditions^5,6^. Existing machine learning applications in epilepsy have primarily focused on using numeric data values such as electroencephalogram (EEG) recordings and imaging data for seizure detection tasks^7–10^. However, a rich set of data elements are available in the patient registries, Electronic Health Record (EHR) systems, and clinical notes describing molecular, pathological, surgical, and laboratory findings, which have not been widely used in machine learning workflows due to feature engineering challenges. A machine learning workflow can be conceptualized with three primary components: (1) input data; (2) feature engineering that creates representations of the input data for use by machine learning models; and (3) mathematical models that generate new insights from the data^11^. Feature engineering involves transformation of raw data into learning features by preprocessing data into appropriate format that can be used for characterizing feature importance and feature interaction, among other tasks; therefore ontologies have an important role in feature engineering tasks^12^.

Biomedical ontologies play a central role in harmonizing disparate datasets for precision medicine, querying large-scale EHR data, and performing multi-dimensional analysis^13,14^. Biomedical ontologies have been widely adopted to reconcile terminological heterogeneity, for example Gene Ontology (GO)^15^, the Systematized Nomenclature of Medicine Clinical Terms (SNOMED CT)^16^, and RxNorm for clinical drug names^17^. Since 2012, we have been developing the Epilepsy and Seizure Ontology (EpSO), which is currently the largest open-source epilepsy-focused ontology, to support comparative analysis of patient record data, differential diagnosis among other applications^18–21^. In addition to these traditional ontology applications, EpSO, as a rigorously designed ontology, has a key role in enabling machine learning workflows to access large volumes of heterogeneous epilepsy clinical data through ontology-based feature engineering.

**Figure 1:**
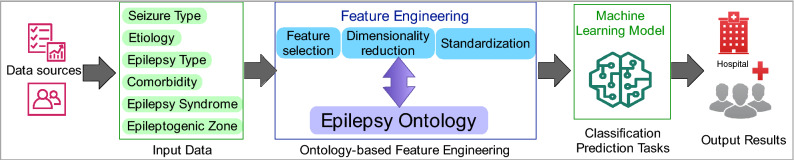
Visualization of a machine learning workflow and the role of epilepsy ontology in feature engineering.

In this study, we expanded and validated the use of EpSO for feature engineering task in a machine learning workflow using three learning models for multilabel multiclass classification of neuropathology reports with diagnosis as output and using immunohistochemistry, microscopy, imaging, and anatomy as input features (Fig. 1).

In the rest of the paper, we describe the context of this work with relation to existing methods in feature engineering using ontologies. In the “Method” section, we describe the details of the engineering approach used to develop the epilepsy ontology and implementation of the ontology-driven generation of features for learning models. We present the results of a comparative evaluation of the effectiveness of ontology-based mappings in machine workflows in the next section followed by discussion and conclusion.

### Research in context: related work and implications of the current study

We performed a keyword-based search in PubMed on February 11, 2022, using the terms “ontology machine learning feature engineering epilepsy” and our search yielded no results. We modified our search by removing the term “epilepsy” with two query expressions with and without the AND logical connective, that is, “ontology machine learning feature engineering” and “ontology” AND “feature engineering” AND “machine learning”. The search query yielded 89 and 5 results respectively with only one paper by Garla et al. describing the use of the Unified Medical Language System (UMLS) as an ontology structure for feature ranking in text classification^22^. Two other papers described the use of controlled terminology for computing semantic similarity in opinion mining from movie reviews and a review paper describing the use of ontologies for feature selection^23,24^. In contrast to these applications, the method described in this paper performs feature engineering in terms of transforming raw input data into learning features, which has a direct impact on both the accuracy as well as the run time performance of the machine learning models. In summary, the new method described in this paper:

1. Performs input data transformation to ontology-based learning features, which is distinct from feature ranking and feature selection applications described in previous work.
2. Implements a systematic three-step ontology-mapping process that uses the formal semantics of the ontology to generate context-aware features (described in the “Method” section). This approach has not been described in any published paper and generating learning features from epilepsy clinical data is a unique challenge that has been addressed in this study.
3. Presents an ontology-driven approach to bridge the differences between two widely used epilepsy and seizure classification systems for improving feature generation from epilepsy clinical data (described in the “Discussion” section).

To our knowledge, this is the first study to investigate the use of ontology for feature engineering in multi-label multi-class classification of epilepsy patient records using non-numeric clinical data. This is also the largest study that analyzes the potential of using compositional ontology class expressions and semantic transformations to create learning features in machine learning workflows. This study provides an assessment of the importance of individual learning features and the impact of ontology-based feature engineering on the performance of machine learning models. In the long term, this ontology-based feature engineering approach is likely to enable machine learning workflows to access large volumes of epilepsy clinical data in EHR systems and patient registries beyond numeric data such as EEG recording for classification and prediction tasks. This feature engineering approach also improves the performance of machine learning models applied to epilepsy data and expand the application of existing biomedical ontologies to machine learning workflows.

## Method

### Study design and participants

We performed a retrospective, proof-of-concept study using de-identified records from the German Neuropathology Reference Center for Epilepsy Surgery at Erlangen, Germany. The study cohort included 315 patients who underwent epilepsy surgery and were diagnosed with dual pathology (*n* = *5*), hippocampal sclerosis (HS) (*n* = *36*), noHS (*n* = *10*), malformations of cortical development (MCD) (*n* = *136*), brain tumor (*n* = *81*), gliosis (*n* = *20*), encephalitis (*n* = *11*), cyst (*n* = *3*), encephalopathy (*n* = *2*), cavernoma (*n* = *2*), Alzheimer’s Disease (*n* = *1*), arteriovenous malformation (*n* = *1*) and otherwise not specified (NOS) (*n* = *7*). Three of the patients were excluded from the study due to the lack of neuropathology diagnosis values in their records; therefore, 312 patients were included in the final analysis. The ground truth for the diagnosis was the original finding recorded in the reports.

We obtained written informed consent from all participating patients or their legal guardians for surgical tissue and clinical data collection in the European Epilepsy Brain Bank (EEBB) hosted at the Department of Neuropathology, Universitätsklinikum Erlangen, which includes the use of tissue and clinical data in medical and scientific investigations, and publication of the results. The Ethics Committee of the Medical Faculty of the Friedrich-Alexander University (FAU) Erlangen-Nürnberg, Germany, approved the study (AZ 160_12B, AZ 92_14B, AZ 193_18B), and all research was performed in accordance with the Declaration of Helsinki.

### Modeling epilepsy neuropathology using ontology engineering methods

As part of the ILAE big data–open data task force, we formed an international collaborative team of neuropathologists, epileptologists and computer scientists, and this team held regular, bi-weekly remote meetings over 18 months between 2020–2021 for this study. We used peer-reviewed publications and a textbook on surgical neuropathology of focal epilepsies by Blumcke et al.^25^, together with both formal as well informal feedback from domain experts outside of the task force for ontology modeling decisions. We focused on modeling four neuropathology topics, that is, HS, MCD, brain tumors, and encephalitis together with immunohistochemistry, microscopy, anatomy, genetics, and imaging terms, which were needed for feature engineering in the machine learning workflow used in this study.

#### Modeling hippocampal sclerosis

HS is a prototypic focal epilepsy syndrome, and the most common cause of temporal lobe epilepsy. Correctly identified and investigated, it is also one of the most surgically remediable syndromes. HS is histopathologically characterized by specific patterns of neuronal cell loss and gliosis within hippocampal subfields. The most common subtype, the classical HS (HS ILAE Type 1; 60–80%) refers to severe neuronal cell loss and gliosis predominantly in cornu ammonis’s (CA) sectors CA1 and CA4, compared to CA1 predominant neuronal cell loss and gliosis in HS ILAE type 2 (5–10% rate of occurrence), or CA4 predominant neuronal cell loss and gliosis in HS ILAE type 3 (4–7% rate of occurrence)^25^. Surgical hippocampus specimens obtained from patients with Temporal Lobe Epilepsy (TLE) may also show normal content of neurons with reactive gliosis only (no-HS).

**Figure 2:**
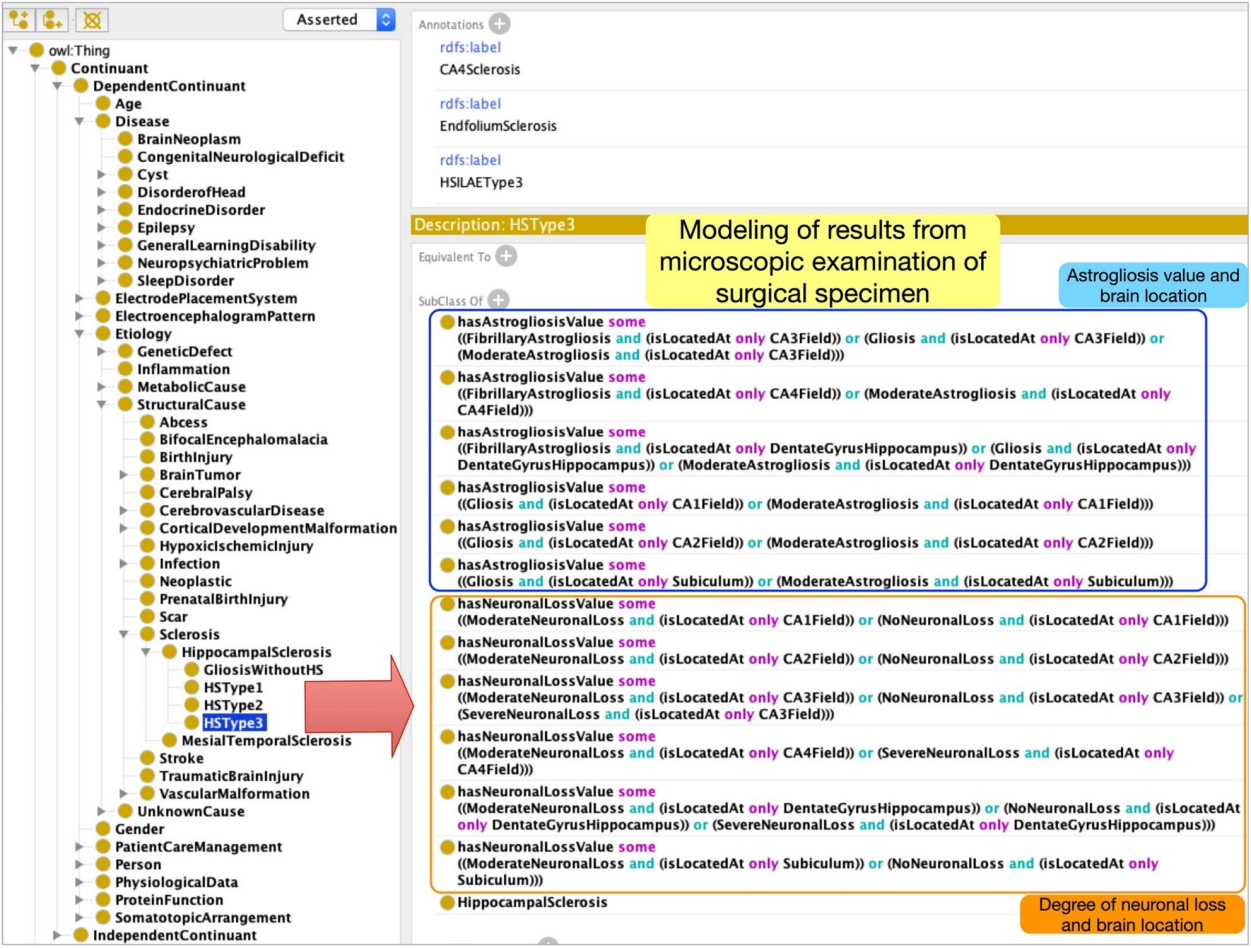
EpSO uses a compositional modeling approach to represent multiple dimensions of neuropathology findings using description logic quantifiers together with OR, AND connectives.

In EpSO, we used the international consensus classification system developed by the ILAE to describe the type of astrogliosis (e.g., moderate astrogliosis or fibrillary astrogliosis) and the level of neuronal loss in specific locations (e.g., *CA1*–*CA4*, dentate gyrus)^25^. EpSO models the four subtypes of hippocampal sclerosis: *HSType 1*, *HSType 2*, *HSType 3*, and *Gliosis without hippocampal sclerosis* (we use italics to distinguish ontology terms in EpSO from clinical terms). However, instead of modeling a large number of subclasses of these terms corresponding to their neuronal loss or astrogliosis values, such as “HSType1 with fibrillary astrogliosis in CA2”, we used a flexible “compositional modeling” approach using existential and universal quantifiers defined over OWL object properties^26^. Figure 2 shows the modeling of *HSType3* with details of *astrogliosis* and *degree of neuronal loss* in specific locations, such as *CA3*, *CA4* and *dentate gyrus*. The brain location terms are modeled as ontology classes in EpSO and mapped to the comprehensive Foundational Model of Anatomy (FMA) ontology with information about their organization based on brain segments and synonymous terms^27^.

The ILAE international consensus classification system assigns numeric values between 0 and 2 based on the type of *astrogliosis* and *degree of neuronal loss*^28^. This semiquantitative grading system was introduced to provide a simplified but practical method of assessing neuronal loss as quantitative neuronal density measurements are time consuming and are often not available despite being more accurate. The descriptive assessment ranges include no obvious neuronal loss or moderate astrogliosis (value = 0), moderate neuronal loss and gliosis (value = 1), and severe neuronal loss (majority of neurons lost) and fibrillary astrogliosis (value = 2) in hippocampal subfields CA1–CA4. In the dentate gyrus the grading is used to describe additional histopathological patterns of either granule cell dispersion (value = 1) or severe granule cell loss (value = 2) that can be found in about 50% of cases with hippocampal sclerosis or a normal dentate gyrus (value = 0). EpSO explicitly models these numeric values associated with the different categories of *astrogliosis* and *degree of numeric values*, which can be used for classification, ranking, and computation of similarity scores between patient records.

#### Modeling malformations of cortical development

These are an important category of intractable epilepsy, with strong genetic underpinnings. Many types of MCD are amenable to surgical interventions, and thus neuropathological tissue analysis is a standard practice. EpSO models 11 subtypes of MCD, including *Polymicrogyria*, *Schizencepahly*, with additional subcategories of *focal cortical dysplasia* (*FCDType 1*, *FCDType 2*, and *FCDType 3*), and *heterotopia* (e.g., *Nodular heterotopia*, *Band heterotopia*, and *cortical dyslamination*). The modeling of staining results in the ontology, for example detection of *Balloon Cell* using *Vimentin* or *Phosphor-S6 Ribosomal Protein epitopes* in *Focal Cortical Dysplasia Type IIB* was a significant engineering challenge. To address this challenge, we developed and used OWL object properties such as *hasNeuroPathologyFinding* together with object property restrictions that were linked with distinct classes of *epitopes* (n = 53) and cell types (Fig. S1 in supplementary document).

The protein functions of the epitopes were mapped to the existing Protein Ontology with their function modeled as a restriction on object properties, for example *Glutamate Decarboxylase 65* (*GAD65*) is involved in *neurotransmitter synthesis*. This interlinking of EpSO terms to external resources is an important ontology engineering best practice that enables interoperability across ontologies. The ILAE consensus neuropathological classification system for focal cortical dysplasia was used as the reference for modeling the relevant terms in EpSO^25^. For example, the co-located neuropathology findings such as occurrence of *cortical dyslamination* adjacent to *vascular malformation* were modeled for *Focal Cortical Dysplasia Type IIIC* using an existential quantifier with the *AND* logical connective.

#### Modeling of brain tumors associated with epilepsy

Tumoral epilepsy is a common finding, and tissue diagnosis is important for accurate classification of tumors, as well as prognostication and outcomes from treatment interventions. Although any brain tumor based on their anatomical location may cause epilepsy, the majority of epilepsy-associated tumors are benign, mainly of neuronal or mixed glial-neuronal origin, and are frequently located in the temporal lobe^25^. Characteristic entities comprise the ganglioglioma, the dysembryoplastic neuroepithelial tumor, and low-grade neuroepithelial tumors like the angiocentric glioma, which together account for the vast majority of tumors identified in retrospective surgical epilepsy case series^25^. In EpSO, our objective was to model the different categories of brain tumors based on their phenotypes, including tumors with predominant glial-neuronal phenotypes such as *ganglioglioma* and *astrocytic* phenotype including *glioma*. Table S1 in the supplementary materials lists EpSO ontology classes corresponding to the seven categories of brain tumors namely *Brain Glial Neuronal Tumor*, *Brain Glial Tumor*, *Brain Neuronal Tumor*, *Hamartoma*, *Epithelial Cyst*, *Meningioma*, and *Metastatic Tumor* together with their subcategories as well as the associated World Health Organization (WHO) grading and gene mutation information^29,30^.

The increasing focus on epilepsy genetics as part of the wider precision medicine (PM) initiative in epilepsy is marked by an increased understanding of the pathogenic variants in genes and how the gain or loss of function mutations result in specific phenotypes^31^. For example, mutation in *GLI3* gene is associated with *Hypothalamic Epilepsy* with an etiology of *Hypothalamic Hamartoma*. Similarly, *Dravet Syndrome* is associated with a loss of function mutation in *SCN1A*, which has multiple sequence variants as listed in the National Center for Biotechnology Information (NCBI) ClinVar database^32,33^. ClinVar is a public database with records of human genetic variations and the associated phenotype that also stores the evidence associated with the reported association.

EpSO models the genetics of epilepsy by linking gene classes with: (1) the NCBI Gene database; and (2) the sequence variants of the genes in ClinVar database with details of the molecular consequence, phenotype, and variant type of the gene. For example, *SCN1A variant* with *variation ID 68500* is linked to its phenotype *Dravet Syndrome*, *Missense variant* as its molecular consequence, and *single nucleotide variant* as its variant type. The modeling of epilepsy genetics terms in EpSO is aimed to facilitate its continued role in semantic integration of medical data, which increasingly feature genetic variations and their role in epilepsy phenotype (Table 1).

**Table 1:** Ontology metrics including classes representing epilepsy related genes.

Ontology metrics (EpSO version 2.1 available at the National Center for Biomedical Ontologies, BioPortal)	Count
Total logical axioms	2592
Class count	1957
Property count (OWL object and datatype properties)	43
Annotation axioms	3318
Gene class count	154

#### Feature engineering using epilepsy ontology

We selected microscopy, imaging results, immunohistochemistry, and anatomical locations as input features to three machine learning models, which assigned one or more neuropathology diagnosis labels to each of the patient records. The raw data elements from the 312 neuropathology reports featured significant terminological heterogeneity with 1328 distinct terms used to describe both input features and output diagnosis labels. The occurrence of a relatively high number of learning features in comparison to the size of dataset is a common challenge in machine learning workflows, which often leads to overfitting and low generalization of the trained model.

**Figure 3:**
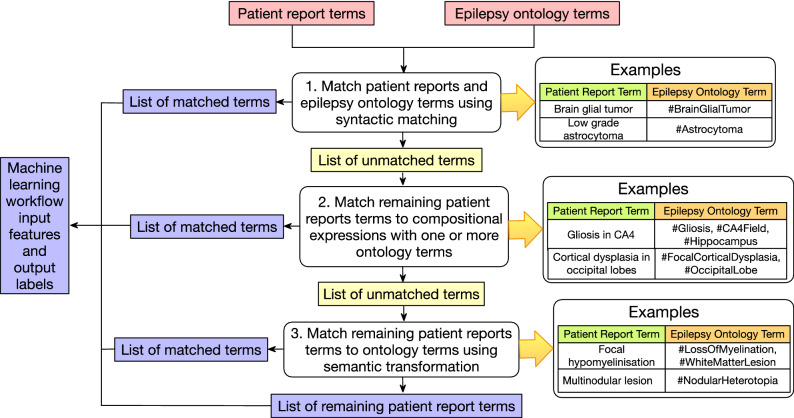
Feature engineering workflow used to map terms in patient reports to epilepsy ontology with a three-step approach. The first step uses syntactic matching, followed by mapping a composition of ontology terms, and finally semantic transformation. All the mappings were manually reviewed. The final list of terms after the three-step matching process is used as input features and output labels to the machine learning models.

Therefore, feature engineering approaches, including the use of embeddings for dimensionality reduction, play an important role in machine learning workflows. In this study, we leveraged the detailed modeling of neuropathology terms in EpSO for feature standardization and for the reduction in variability across both input as well as output features. The ontology-driven feature engineering was implemented manually (Fig. 3) and consisted of three approaches:

1. Multiple terms mapped to a standard ontology term: For example, terms “microglia nodules”, “periventricular nodular heterotropia”, “multinodular lesion”, “Heterotropic neuronal nodules at periventricular site”, and “Bilateral periventricular heterotropia” were mapped to *Nodular Heterotropia*.
2. A term mapped to composition of ontology terms: For example, “depletion of neuron in CA2”, “segmental cell loss in CA2”, “neuronal cell loss in CA2”, “reduced neuronal density in CA2” were mapped to a composition of *Neuronal Loss* and *CA2*.
3. Semantic transformation of term to map to ontology terms: For example, “astroglial phenotype” was mapped to composition of *GlialCell*, *Astrocyte*, and *BrainGlialTumor*.

Additional details of the ontology-based feature engineering are described in supplementary document Section 1.1. Table 2 shows the results of mapping the original list of features extracted from the neuropathology reports and the number of EpSO classes that were mapped to these features. All the mappings were reviewed by a neuropathologist for consistency and accuracy.

**Table 2:** Result of feature engineering using mappings to EpSO terms.

	Original number of terms in patient reports	Number of terms after ontology mapping	Decrease in the number of terms (in %)
Microscopy	802	125	84.41
Immunohistochemistry	141	84	40.4
Imaging results	218	82	62.38
Anatomical location	167	61	63.47
Diagnosis	149	80	46.30

We used machine learning libraries from the open source Scikit library^34^, and the details of the model architecture, parameters, binary relevance transformation method for multilabel classification, and validation methods are described in supplementary document Section 1.2.

## Results

### Comparative evaluation of ontology-based feature engineering

We compared the impact of ontology-based feature engineering on the three machine learning models with baseline results computed without any mapping to ontology terms. In the first stage, only input features were mapped to ontology terms and in the second stage, both input features as well as output labels were mapped to ontology terms. The results (in Table 3) are categorized into two types: (1) correct results, where the diagnosis results of a machine learning model match the ground truth (original diagnosis in patient reports); and (2) partially correct results, where the diagnosis results consist of a subset of the ground truth diagnosis terms; which may include incorrect diagnosis labels.

**Table 3:** Comparative evaluation of ontology-based feature engineering in the logistic regression, random forest, and gradient boosting tree models.

	Logistic regression			Random forest			Gradient tree boosting		
	Baseline	Mapping ontology terms to input features	Mapping ontology terms to input and output features	Baseline	Mapping ontology terms to input features	Mapping ontology terms to input and output features	Baseline	Mapping ontology terms to input features	Mapping ontology terms to input and output features
Correct results	14	17	19	11	16	17	9	10	12
Partially correct results	45	41	37	50	44	43	40	42	41

The values reported in Table 3 are based on the result of a five-fold cross validation process. The logistic regression, random forest, and gradient tree boosting models show 35.7%, 54.5%, and 33.3% improvements in the generation of correct results respectively when both input features and output labels were mapped to ontology terms. The results also show that even partial mapping of input features to ontology terms leads to improvement across all the three machine learning models, including reduction in the mixed results category that consists of both correct as well as incorrect diagnosis terms.

### Evaluation of ontology-mapping on individual learning features

**Figure 4:**
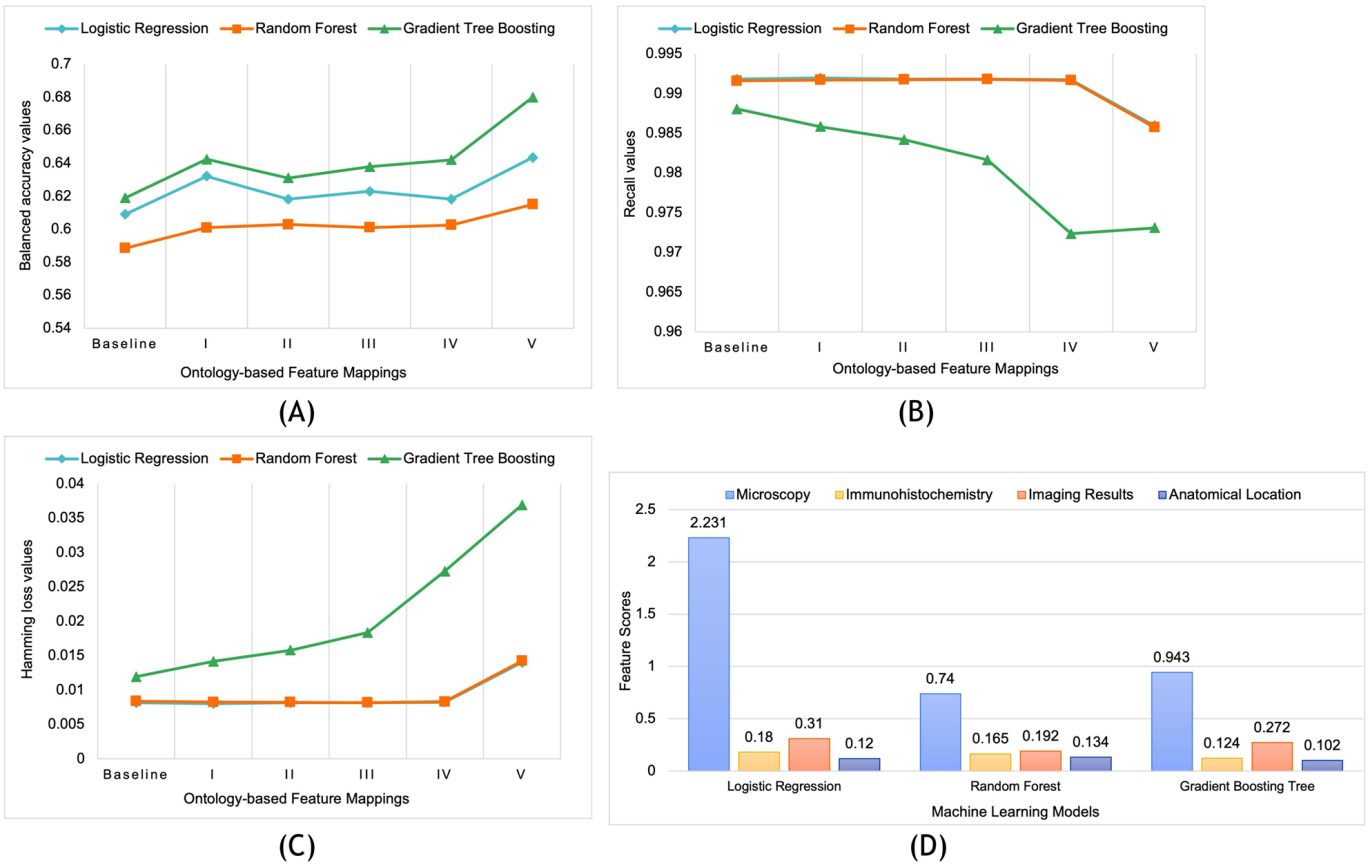
Comparative evaluation of the effect of ontology-based feature engineering using three metrics of balanced accuracy (**A**), recall (**B**), and hamming loss (**C**), for all three machine learning models. The baseline values do not have any ontology mappings and Cases I to V correspond to the addition of ontology mappings to Microscopy, Immunohistochemistry, Imaging results, Anatomical location, and Diagnosis (output label) respectively. (**D**) Feature importance score of the input features that affect the performance of the three machine learning models.

We used three common performance metrics for multilabel multiclass classification tasks, namely Hamming loss, balanced accuracy, and recall to evaluate the effect of ontology mappings on individual features^35^ (details of the three metrics are described in supplementary document Section 1.3). Figure 4 shows the performance of the three models as each of the four input features and output feature are progressively mapped to ontology terms. The accuracy values (Fig. 4A) improve as an increasing number of input features are mapped to the ontology terms with mappings to both input features and output labels showing the highest improvement. We note that there is a marginal decrease in recall values as an increasing number of input and output features are mapped to ontology terms thereby reducing the total number of features (Fig. 4B).

However, we note that the accuracy and recall values do not consistently increase or decrease as individual input features are mapped to ontology terms. For example, the mapping of immunohistochemistry and brain anatomical region terms to ontology terms results in a decrease in accuracy values for logistic regression model, whereas the mapping of these two categories of input values to ontology terms results in an increase in accuracy values for random forest model. This pattern is also seen for recall values in all the three machine learning models. Figure 4C shows that there is improvement in hamming loss values as microscopy and immunohistochemistry terms are mapped to EpSO for logistic regression and random forest models; however, the performance of the three models measured by hamming loss decreases as diagnosis values are mapped to ontology terms.

### Importance of individual learning features

To investigate these variations in performance of individual features after ontology mappings, we evaluated the contribution of each of the features to the classification task. There are multiple approaches for measuring feature importance that estimate the importance of a feature to provide an improved understanding of how machine learning models use input features to generate results^36,37^. Feature importance scores are used in feature engineering to address bias, and allow users to interpret results in terms of the contribution of individual input features^38^. We used the Scikit-learn libraries to compute feature importance scores for each of the four input features without any ontology mappings for all the three models (Fig. 4D). The results show that the input feature microscopy has a consistently high feature importance score across all three machine learning models. We note that applying an ontology-based feature engineering approach on microscopy terms resulted in the highest reduction in total number of terms (84.41% in Table 2), which combined with its high feature importance score may be correlated to the improvement of balanced accuracy measure across all three learning models as shown in Fig. 4D.

In contrast, the feature importance score for immunohistochemistry is low across all the three models and we also note that the use of ontology mappings resulted in the lowest reduction of terms (40.4% in Table 2) for this input feature (labeled as Case II in Fig. 4D). The feature importance score of imaging is relatively high across all the three models whereas the score for the anatomy feature is the lowest. These feature importance scores provide an overview of the contribution of each of the input features to the performance of the three machine learning models. However, these scores are not adequate to characterize the interactions between different features and their impact on the performance of the machine learning models. For example, the importance of individual features such as microscopy and combined features such as imaging results and anatomy requires additional evaluations over a larger dataset^12,39^. A detailed evaluation of feature interaction and the feature importance analysis could enable us to explore additional feature engineering steps, such as decomposing anatomy into features based on brain lobes or immunohistochemistry values into individual features based on epitope target (e.g., proliferation index or marking of cellular patterns).

### Impact of ontology-based feature engineering on run time performance of machine learning models

**Figure 5:**
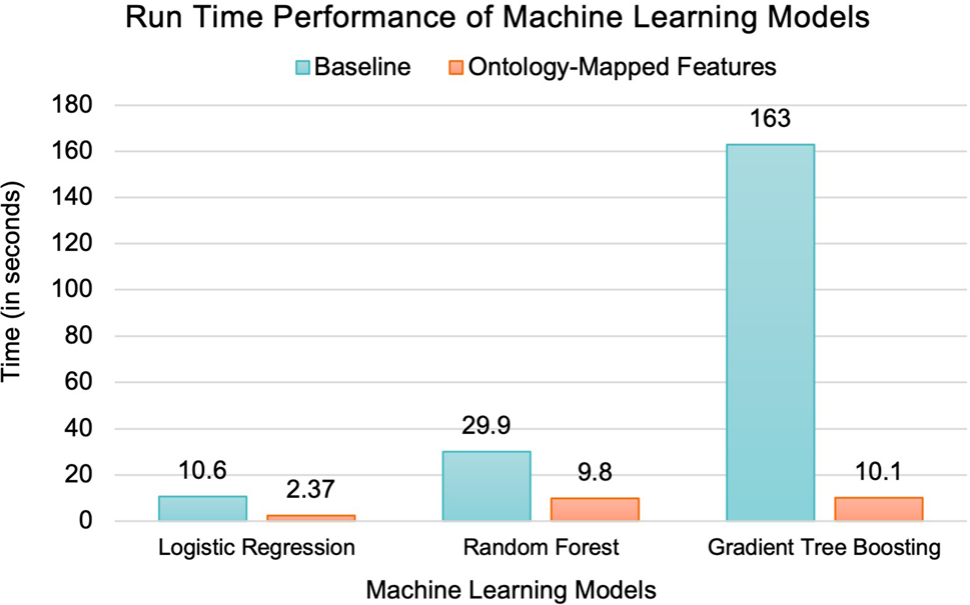
Run time performance of the three machine learning models without ontology-based mappings (baseline) and with mappings of both input as well as output values.

There has been significant focus on the run time performance of machine learning implementation, including the use of specialized hardware such as Tensor Processing Units (TPU) or Graphics Processing Unit (GPU), for reducing the time required to execute machine learning workflow^40,41^. In this study, we evaluated the effect of ontology-based feature engineering on the run time performance of the implemented workflow, which showed significant improvement across all three machine learning models. The tests were performed on a server with 32 GB memory, Intel® Core™ i7-9700K CPU (3.60 GHz × 8) processor running 64-bit Ubuntu 20.04.4 LTS. The results were based on the average of seven executions. Figure 5 shows that there is an improvement of 93.8% for gradient tree boosting, 67.2% for random forests, and 77.6% for logistic regression with ontology-based feature engineering as compared to the baseline. It is important to note that the reduction in run time of all the three models corresponded to consistent improvement in the balanced accuracy of all three machine learning models. A key reason for the significant impact of ontology-based feature engineering on run time performance of the learning models may be due to the standardization of input features using the epilepsy ontology, and we propose to develop a benchmark evaluation to characterize this effect in our future work.

## Discussion

### Reconciling heterogeneous epilepsy and seizure classification system in epilepsy ontology

The ILAE classification system (ILAE-EC) and the four-dimensional classification system (4D-EC) are two widely used classification systems for epilepsies and seizure^5^. Due to the inherent complexity of epilepsy as a heterogeneous condition, the use of two classification systems further exacerbate data harmonization and feature engineering challenges in machine learning workflows. A recent paper by Rosenow et al. proposed to use five common axes of seizure type, etiology, epilepsy type, comorbidity, epilepsy syndrome, and epileptogenic zone to reconcile the ILAE-EC and 4D-EC classification systems^5^; however, there were no existing implementations of the proposed approach. We implemented the proposed approach in EpSO that can support feature engineering over datasets using either of the two commonly used classification systems.

**Figure 6:**
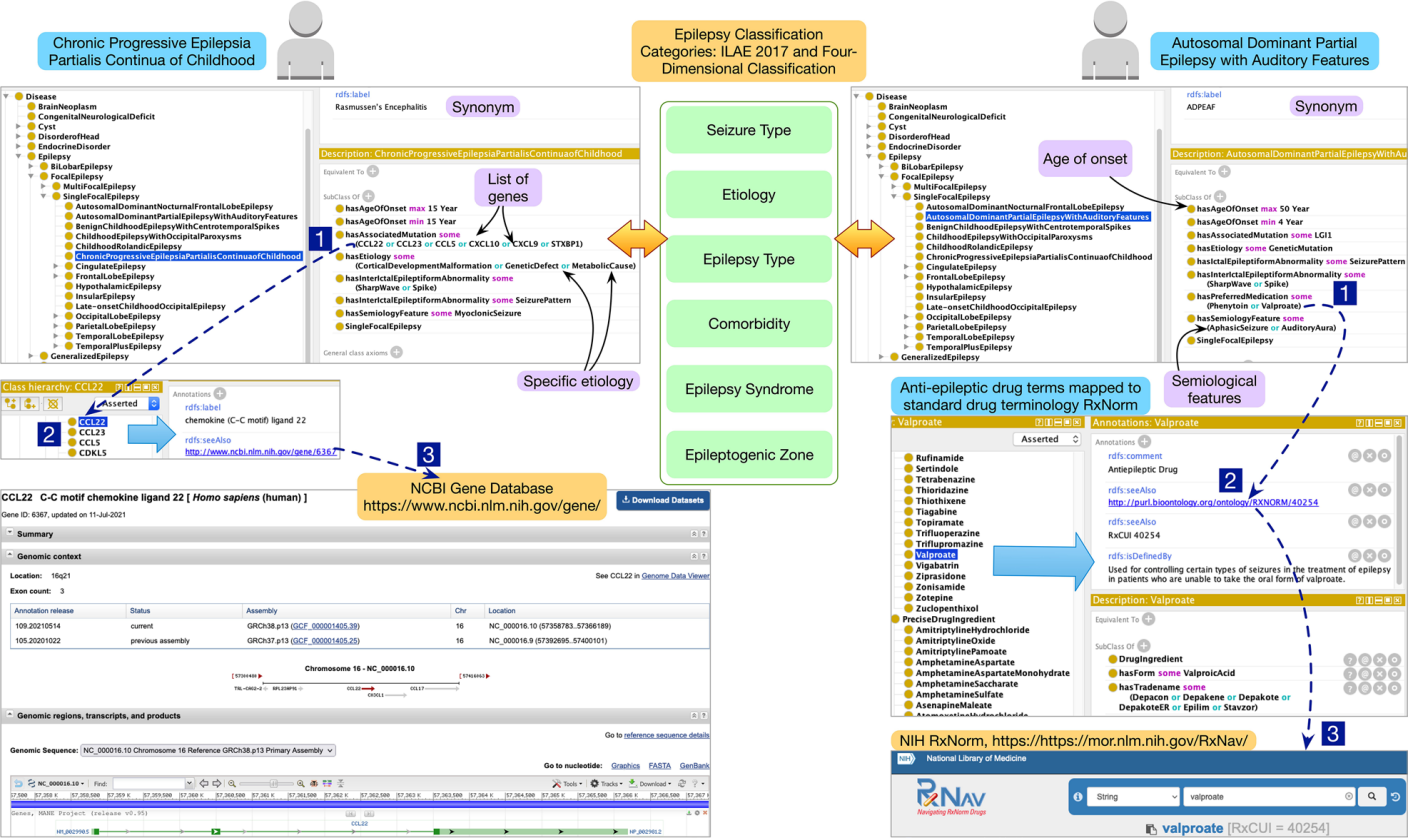
Modeling of two epilepsy syndromes in EpSO that includes attributes based on both ILAE 2017 classification system and the four-dimensional classification system.

Figure 6 shows the modeling of two epilepsy types (*Chronic Progressive Epilepsia Partialis Continua of Childhood* and *Autosomal Dominant Partial Epilepsy with Auditory Features*) with a set of attributes mapped to ILAE-EC and 4D-EC. These ontology mappings in EpSO are accessible to software tools for automated parsing; therefore, applications that use EpSO terms can also identify specific ILAE 2017 seizure types associated with the ontology term. Ontology properties defined in the OWL specifications^26^ allow us to flexibly update the mappings of ontology terms to reflect future revisions that may be proposed by the ILAE-EC and these updates can also be automatically propagated to machine learning workflows through the use of embedding libraries. These embedding libraries with ontology mappings could be made available for reuse across machine learning workflows through version control platforms such as GitHub.

### Ontology-based feature engineering with support for explainable AI

The computation of feature importance scores and quantifying the interactions between features are important steps to help understand the results generated by machine learning models, including the association of reliability score that can be shared with users^36,37^. There are multiple frameworks for explaining the performance of machine learning models, including interpretability frameworks for tree-based models, such as random forests and gradient boosted trees, using game theory to explain the effect of input features on a single output result^36,37^. Some of these frameworks are based on the path used by a model to generate an outcome, which can be augmented by the interlinked structure of an ontology. Further, the interaction between different features can also be characterized using reasoning algorithms that traverse the ontology structure using *isA*, *part of*, *hasEtiology*, and other ontology properties as modeled in EpSO. The use of description logic-based reasoning algorithms could improve the interpretability of results generated by different models. However, this proposed approach will require addressing the challenges of bias in mapping of input features to ontology terms, including the use of multiple ontology classes to represent a single input feature.

### Limitations

This study is limited to a single site dataset and the selection of patient reports was based on a criterion that the reports were available in English; therefore, the study cohort does not address bias in terms of demography, clinical findings, and subcategory of epilepsy patients who were considered for neuropathology evaluation. The mapping approach used in this study was verified by a single neuropathologist, which may lead to bias in feature generation and the corresponding results generated by the machine learning models. The feature importance metric used in this study does not account for feature interactions and any correlation between the features.

In conclusion, our findings demonstrate that ontology-based feature engineering is effective in improving the performance of learning models and it can be used to unlock the value of large volumes of heterogeneous epilepsy clinical data in patient registries and EHR systems. As a next step, we plan to expand this study to multi-institution datasets and apply deep neural network models together with Shapely values for explainable results that may be integrated into clinical decision support systems.

## Supplementary Information

Supplementary Information.

## Data Availability

The ontology was engineered using the open source Protégé ontology development application. The new version of EpSO (version 2.1) was released through the National Center for Biomedical Ontology BioPortal portal in October 2021 (https://bioportal.bioontology.org/ ontologies/EPSO). The machine learning workflows and performance metrics were implemented using the Scikit libraries. The individual patient records cannot be made publicly available due to regulatory reasons. Models and data can be made available on request; however, this requires the execution of a data transfer agreement approved by the participating institutions together with an Institutional Review Board (IRB) or equivalent ethics approval for the proposed study.
