# Supplementary material for: Ontology-based feature engineering in machine learning workflows for heterogeneous epilepsy patient records
Source: Sci Rep. 2022 Nov 12;12:19430. doi: 10.1038/s41598-022-23101-3 (PMC9653502; doi:10.1038/s41598-022-23101-3)
Supplement: Supplementary file 1 — Supplementary Information. [file 41598_2022_23101_MOESM1_ESM.pdf]

# Supplementary Material

## Ontology-based feature engineering in machine learning workflows for heterogeneous epilepsy patient records

Satya S. Sahoo, Katja Kobow, Jianzhe Zhang, Jeffrey Buchhalter, Mojtaba Dayyani, Dipak P. Upadhyaya, Katrina Prantzalos, Meenakshi Bhattacharjee, Ingmar Blumcke, Samuel Wiebe, Samden D. Lhatoo

**Contents:**

1. Supplementary Methods
2. Supplementary Table and Figures

### Supplementary Methods

Here we present additional details of the method used in using ontology-based feature engineering, implementation of the machine learning workflow, and the evaluation metrics used in the study.

#### 1.1 Feature engineering using epilepsy ontology

As part of the three-step feature engineering process used to map values in the patient records to ontology terms, we first used syntactic mappings based on synonyms and related annotation properties (modeled using *rdfs:label* and *rdfs:comment* in the ontology) (1-3)). The syntactic transformation involves removal of whitespace in a phrase (e.g., "Mesial Temporal Sclerosis") or mapping specific parts of the phrase ("Atypical Ganglioma WHO grade II" to *AtypicalGaglioma*) as the associated WHO grading is already modeled in EpSO using quantifier restriction on the object property *hasWorldHealthOrganizationGrading*. In the second step, we used class expressions that combined one or more ontology terms to represent a complex term.

This composition-based class expressions using one or more ontology terms for representing medical concepts has also been implemented in the Systematized Nomenclature of Medicine Clinical Terms (SNOMED CT) (4). SNOMED CT uses a combination of pre coordination, where terms are modeled explicitly in an ontology (precoordinated expressions), and post coordination, where one or more terms are combined using a set of rules (post coordinated expressions). The use of post coordinated expressions in SNOMED CT enables it to model new medical concepts or terms. For example, a SNOMED CT post coordinated expression using the recommended syntax |*hip joint*| : |*laterality*| = |*left*| represents the laterality information about a hip joint (4).

In this study, we used an aggregation of epilepsy ontology terms to map a value in the patient record; for example, "depletion of neuron in CA2" was mapped to *NeuronalLoss*, *CA2Field*, *PyramidalNeuron*, and *HippocampalSclerosis*. We note that the first three ontology terms together model the term from the patient record and the fourth term describes important contextual information (diagnosis information), which is important in a machine learning workflow. A similar term augmentation occurs when the patient record term "Blurring" is mapped to the ontology term *BlurringOfGreyWhiteMatterJunction*, which provides additional contextual information.

In the third step of the feature engineering step, we used semantic transformation approach that uses the semantics of a patient record term for mapping to an ontology term. For example, the term "microcolumn" was mapped to *AbnormalRadialCorticalLamination*, *FocalCorticalDysplasiaTypeIA*, and *OccipitalLobe* by interpreting the occurrence of microcolumns to the specific type of cortical dyslamination seen in focal cortical dysplasia type 1A. This mapping considered that although abnormal radial cortical lamination occurs in both focal cortical dysplasia type 1A and focal cortical dysplasia type 1C; however focal cortical

dysplasia type 1C also includes the finding of abnormal tangential cortical lamination. Therefore, “microcolumn” was not mapped to focal cortical dysplasia type 1C.

The mapping of patient record terms to epilepsy ontology terms required manual review and curation; therefore, dissemination of these mappings through a look up service will enable other users to reuse these mappings in their machine learning workflows. As additional mappings are created for new machine learning workflows, a library of these mappings can be a valuable resource for feature engineering of epilepsy clinical data.

## 1.2 Machine learning workflow: parameters, training, and validation

The input features $F_N$ from the 312 neuropathology reports $NP_R$ result in a feature matrix denoted by *FM* ∈ ℜ^(F_N X 〖NP〗_R ) with the diagnosis values used as labels to be assigned to a patient record by each of three models. Therefore, the machine learning task was implemented as multilabel classification based on the binary relevance (BR) transformation method where a patient can have one or more neuropathology diagnosis label (D with |D| = 59) with each label being independent of each other (5, 6). Each of the three models were trained for each diagnosis label based on the four input features, that is, microscopy (M), immunohistochemistry (IHC), brain localization (L), and imaging results (I). For example, neuropathology record with input features M, IHC, L, and I with output D label *Ganglioglioma WHO grade I*. The data values in the reports were encoded using the Scikit “OneHotEncoder” library.

We used the Scikit “liblinear” solver for fitting the logistic regression model with “l2” regularization with a tolerance value of 0.01 for stopping, and relative strength of regularization value set to 1. The random forests library in Scikit is an implementation of the ensemble machine learning method that combines decision trees using random features to improve performance of the model (7). The Scikit random forest library used the “n_estimator” variable to denote the total number of decision trees in the forest, which was assigned a value of 21 during our parameter tuning phase based on the lowest number of incorrect predictions. The Scikit library uses additional parameters to use sample drawn with replacement from the data used for training (also called bootstrap sample), which is set to “true” in our implementation with the generalization accuracy estimated from the left-out samples with the relevant parameter “oob_score” set to true (6). The third model used in this paper is gradient tree boosting with the learning rate parameter set to 0.1, the parameter for the number of weak learners “n_estimator” is set to 31, and the parameter to select the fraction of samples used for fitting the number of individual base learners is set to a value of 0.95. These parameter values are tuned based on the performance of test evaluations performed using a range of parameter values.

To avoid overfitting, we used 5-fold cross validation with each iteration leaving out one subset of data for testing. The trained classifier was used to predict whether each of the diagnosis labels can be assigned to a patient record based on their neuropathology features. This leave-one-out approach takes into consideration our assumption that all the patient reports in our dataset are independent of each other and that the reports were created by a similar process. We used the aggregate of the iterations to generate the final assignment of a diagnosis label to a patient report.

## 1.3 Evaluation metrics: Hamming loss, balanced accuracy, and recall

If N is total number of samples, L is total number of labels, $Y_i$ is the set of true class labels, and $\widehat{Y_i}$ is the set of labels predicted by a classifier *m*, then, the Hamming loss is defined as HL(*m*, N) = $\frac{1}{N}\sum_{i=0}^{N-1}\frac{1}{|L|}\,|\widehat{y_i}\ \Delta y_i|$, where $\Delta$ is the symmetric difference between two sets (5, 6). The accuracy measure is defined as A(*m*, N) = $\frac{1}{N}\sum_{i=0}^{N-1}\frac{1}{|L|}\frac{|Y_i \cap \widehat{Y_i}|}{|Y_i \cup \widehat{Y_i}|}$ (5, 6). The Scikit library includes a specialized function called balanced accuracy that address the issue of bias in imbalanced datasets, and it is computed by assigning a weight to each sample based on the occurrence of the true positive labels (6). In addition to these two metrics, we used recall measure to evaluate the performance of the three models, which is defined as

$\frac{True\ Positive\ (TP)}{True\ Positive\ (TP) + False\ Negative\ (FN)}$, where true positive (TP) corresponds to correct diagnosis labels assigned by a model to a patient record and false positive (FP) corresponds to the correct diagnosis labels that were not assigned by a model to a patient record (8). A lower hamming loss value, higher accuracy value, and higher recall values are indicative of improved performance by a machine learning model.

### 1.4 Statistical analysis of the results.

To validate the significance of our comparison, we conducted a corrected repeated k-fold cv test based on 5 repetitions of 5-fold cross validation (9). A 5-fold cross validation approach was used to calculate the balanced accuracy, hamming loss, and recall for our baseline and class V ontology mapping for each machine learning algorithm. The accuracy measures for each of the 5 folds were recorded. For each algorithm, this 5-fold approach was repeated 5 times, resulting in 25 accuracy measures for each metric for either of the ontology mappings. These accuracy measures were then compared using a t-test using the following formula, where:

(1) r is the number of replications,

(2) k is the number of folds for cross validation,

(3) $a_{ij}$ refers to the accuracy metric (hamming loss, balanced accuracy, or recall) from fold j of replication i for one of the algorithms (random forest, logistic regression, or gradient boosting) for the baseline mapping

(4) $b_{ij}$ refers to an accuracy metric (hamming loss, balanced accuracy, or recall) from fold j of replication i for one of the algorithms (random forest, logistic regression, or gradient boosting) for the class V ontology mapping

(5) $n_1$ refers to the number of instances used for training, and

(6) $n_2$ refers to the number of instances used for testing.

$$t = \frac{\frac{1}{kr}\sum_{i=1}^{k}\sum_{j=1}^{r} a_{ij} - b_{ij}}{\sqrt{\left(\frac{1}{kr} + \frac{n_2}{n_1}\right)\frac{\sum_{i=1}^{k}\sum_{j=1}^{r}\left(a_{ij} - b_{ij} - \frac{\sum_{i=1}^{k}\sum_{j=1}^{r} a_{ij} - b_{ij}}{kr}\right)^2}{kr-1}}}$$

P-values were calculated from the test statistic according to a t-distribution with 24 degrees of freedom (df = kr-1). All calculations were performed in Python (version 3.10).

The results (with p = 0.05) show that the improvement in balanced accuracy is not statistically significant for all the three machine learning models. Similarly, the change in the hamming loss and recall values are also not statistically significant across all the three learning models

## 2 Tables and Figures

**Table S1: Seven subcategories of brain tumors related to epilepsy based on their phenotype**

| Brain Glial Neuronal Tumor | | |
|---|---|---|
| | Anaplastic Ganglioglioma | WHO Grade III |
| | Atypical Ganglioglioma | WHO Grade II **OR** WHO Grade III |

| | | | | | |
|---|---|---|---|---|---|
| | Diffuse Leptomeningeal Glioneuronal Tumor | | | | |
| | Dysembryoplastic Neuroepithelial Tumor | WHO Grade I | Mutation in Gene **FGFR** | | |
| | Ganglioglioma | WHO Grade I | Mutation in Gene **BRAF** | | |
| | Myxoid Glioneuronal Tumor | | Mutation in Gene **PDGFR** | | |
| | Papillary Glioneuronal Tumor | | Mutation in Gene **PRKCA** | | |
| | Rosette-forming Glioneuronal Tumor | | | | |
| Brain Glial Tumor | | | | | |
| | Diffuse Glioma | | | | |
| | | Astrocytic | | | |
| | | | Astrocytoma | WHO Grade II-IV<br>Mutation in Gene **IDH** | |
| | | | Diffuse Astrocytoma | WHO Grade I<br>Mutation in Gene **MYB** **OR MYBL1** | |
| | | | Glioblastoma Multiforme | WHO Grade IV<br>**IDH** wildtype | |
| | | | Diffuse Midline Glioma | WHO Grade III-IV<br>Mutation in Gene **H3** | |
| | | | Diffuse Low Grade Glioma | Mutation in Gene **FGFR** | |
| | | | Diffuse High Grade Glioma | **IDH** wildtype, **H3** wildtype | |
| | | | Angiocentric Glioma | WHO Grade I<br>Mutation in Gene **MYB** | |
| | | Mixed Astrocytic Oligodendroglial | | | |
| | | | Oligoactrocytoma | | |
| | | Oligodendroglial | | | |
| | | | Oligodendroglioma | WHO Grade II-III<br>Mutation in Gene **IDH** **AND**<br>co-Deletion of **1p** **AND** **19q** | |
| | | | Polymorphous low-grade neuroepithelial tumor of the young | WHO Grade I<br>Mutation in Gene **BRAF** **OR FGFR** | |
| | Non-Diffuse Glioma | | | | |
| | | Ependymal Tumor | | | |
| | | | Anaplastic Ependymoma | WHO Grade III | |
| | | | Ependymoma | | |
| | | | Ependymoma RELA-Fusion Positive | WHO Grade III | Gene Fusion **RELA** |
| | | | Myxopapillary Ependymoma | WHO Grade I | |
| | | | Subependymoma | WHO Grade I | |

| | | | | | |
|---|---|---|---|---|---|
| | | Other Astrocytic Tumor | | | |
| | | | Anaplastic Pleomorphic Xanthoastrocytoma | WHO Grade III | Mutation in Gene **BRAF** |
| | | | Pilocytic Astrocytoma | WHO Grade I | Gene Fusion **KIAA1549-BRAF** |
| | | | Pleomorphic Xanthoastrocytoma | WHO Grade II-III | |
| | | | Subependymal Giant Cell Astrocytoma | Mutation in Gene **TSC** | |
| | | | Astroblastoma | Mutation in Gene **MN1** | |
| | | | Isomorphic Astrocytoma | WHO Grade I | |
| Brain Neuronal Tumor | | | | | |
| | Neurocytoma | WHO Grade II | | | |
| | Multinodular and vacuolating neuronal tumor | WHO Grade I | | | |
| Epithelial Cyst | | | | | |
| Hamartoma | | | | | |
| | Hypothalamic Hamartoma | | Mutation in Gene **GLI3** | | |
| Meningioma | | | | | |
| Metastatic Tumor | | | | | |

**Table S2: Mappings between patient records and epilepsy ontology terms categorized by output label (diagnosis) and input features**

| **Diagnosis** | |
|---|---|
| **Patient Record Term** | **Ontology Terms** |
| Brain glial tumor | BrainGlialTumor |
| Ganglioglioma | Ganglioglioma |
| Atypical Ganglioglioma WHO grade I | AtypicalGanglioglioma |
| Focal Cortical Dysplasia Type I | FocalCorticalDysplasiaTypeI |
| Pilocytic astrocytoma WHO grade I | PilocyticAstrocytoma |

| Immunohistochemistry | |
|---|---|
| **Patient Record Term** | **Ontology Terms** |
| GFAP | GlialFibrillaryAcidicProtein |
| MAP2 | MicrotubuleAssociatedProtein2 |
| CD34 | LymphocyteAntigenCD34 |
| Ki-67 | Ki-67Antigen |
| p53 | Phosphoprotein_p53 |

| Microscopy | |
|---|---|
| **Patient Record Term** | **Ontology Terms** |
| Brain glial tumor | BrainGlialTumor |
| Salt and pepper chromatin aggregates | BrainTumor |
| Atypical nuclei | BrainTumor |
| Multi-nucleated cells | BrainTumor, BalloonCells |
| Rosenthal fibres | BrainTumor, PilocyticAstrocytoma, Astrocyte, GlialCell |

| Anatomy | |
|---|---|
| **Patient Record Term** | **Ontology Terms** |
| occipital-basal | Basal, OccipitalLobe |
| temporo-occipital | TemporalLobe, OccipitalLobe |
| left temporo-polar | LeftCerebralHemisphere, TemporalLobe TemporalPole |
| left parietal cortex | LeftCerebralHemisphere, ParietalLobe |
| right temporal lobe | RightCerebralHemisphere, TemporalLobe |

| Imaging Terms | |
|---|---|
| **Patient Record Term** | **Ontology Terms** |
| MRI-positive | WhiteMatterLesion MagneticResonanceImaging |
| Ammon's horn sclerosis | Hippocampus, HippocampalSclerosis |
| mesial tumor | BrainTumor |
| Small lesion | WhiteMatterLesion |
| Left fronto-mesial dysplasia | FocalCorticalDysplasia, LeftCerebral Hemisphere, FrontalLobe |

**Table S3: The p-values for the three evaluation metrics across the three machine learning models.**

| | Gradient Boosting | Random Forest | Logistic Regression |
|---|---|---|---|
| **Balanced Accuracy** | 0.556 | 0.059 | 0.269 |
| **Hamming Loss** | 0.069 | 0.555 | 0.555 |
| **Recall** | 0.069 | 0.555 | 0.555 |

**Table S4: Distribution of diagnosis labels (20 most frequent labels) across 312 patient records**

| Baseline | Frequency (n) | Ontology mapped (Input and Output) | Frequency (n) |
|---|---|---|---|
| Focal Cortical Dysplasia TypeIIB | 33 | Focal Cortical Dysplasia Type IB | 33 |
| Hippocampal Sclesoris TypeI | 22 | Cortical Developmentmal Malformmation | 23 |
| Ganglioglioma WHO grade I | 20 | Hippocampal Sclesoris TypeI | 22 |
| Mild Malformation of Cortical Development, Oligodendroglial | 16 | Ganglioglioma | 20 |
| Reactive gliosis | 9 | Cortical Development Malformation, Oligodendroglial | 16 |
| Dysembryoplastic neuroepithelial tumor WHO Grade I | 9 | Gliosis | 10 |
| Mild Malformation of Cortical Development | 8 | DysembryoplasticNeuroepithelial Tumor | 9 |
| Focal Cortical Dysplasia TypeIB | 8 | Focal Cortical Dysplasia TypeIB | 8 |
| Mild Malformation of Cortical Development Type II | 7 | Astrocytoma | 7 |
| Focal Cortical Dysplasia TypeIIA | 6 | Focal Cortical Dysplasia TypeIIA | 6 |
| Pilocytic astrocytoma WHO I | 5 | PilocyticAstrocytoma | 6 |
| Astrocytoma WHO grade I | 5 | GliosisWithoutHS | 5 |
| Atypical Ganglioglioma WHO grade II | 4 | Mesial Temporal Sclerosis | 5 |
| Mesial Temporal Sclerosis Type Ib | 4 | Glioblastoma Multiforme | 5 |
| Rasmussen Encephalitis | 3 | Scar | 4 |
| Hippocampal sclerosis type I, Focal corticcal Dysplasia TypeIIIA | 3 | Rasmussen Encephalitis | 4 |
| Focal cortical Dysplasia Type IIID, Glial scar | 3 | Encephalitis | 4 |
| Ganglioglioma WHO grade I, Hippocampal sclerosis type I | 3 | Atypical Ganglioglioma | 4 |

| | | | |
|---|---|---|---|
| No Hippocampal sclerosis | 3 | Brain Tumor | 3 |
| Focal Cortical Dysplasia TypeIA | 3 | Focal Cortical Dysplasia TypeIA | 3 |

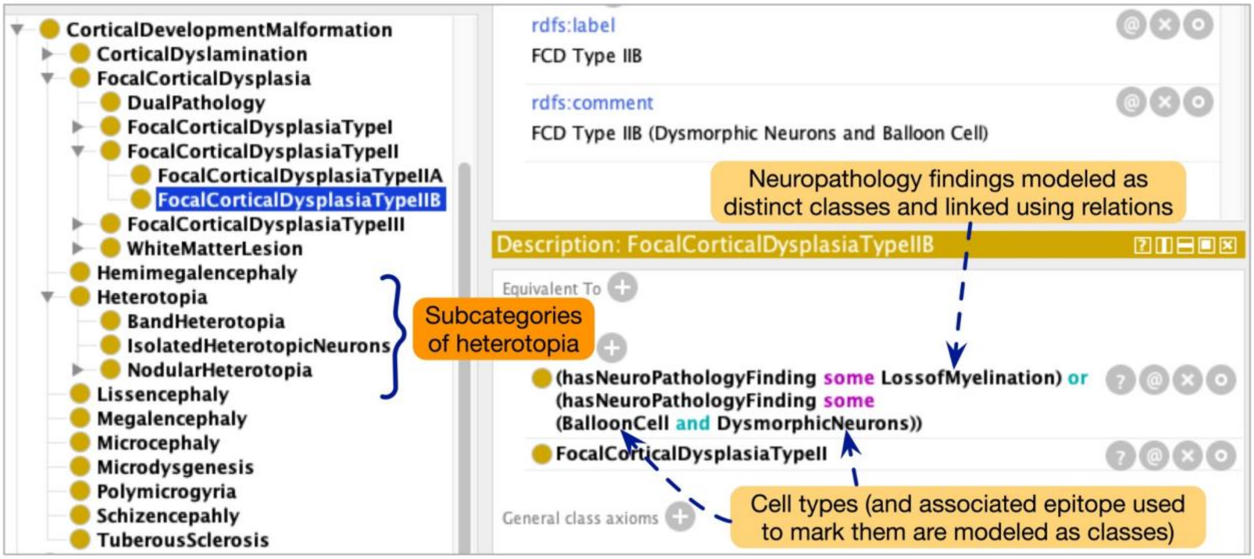


**Figure S1:** EpSO models neuropathology findings at fine level of granularity to support semantic annotation of patient records and applications in feature engineering.

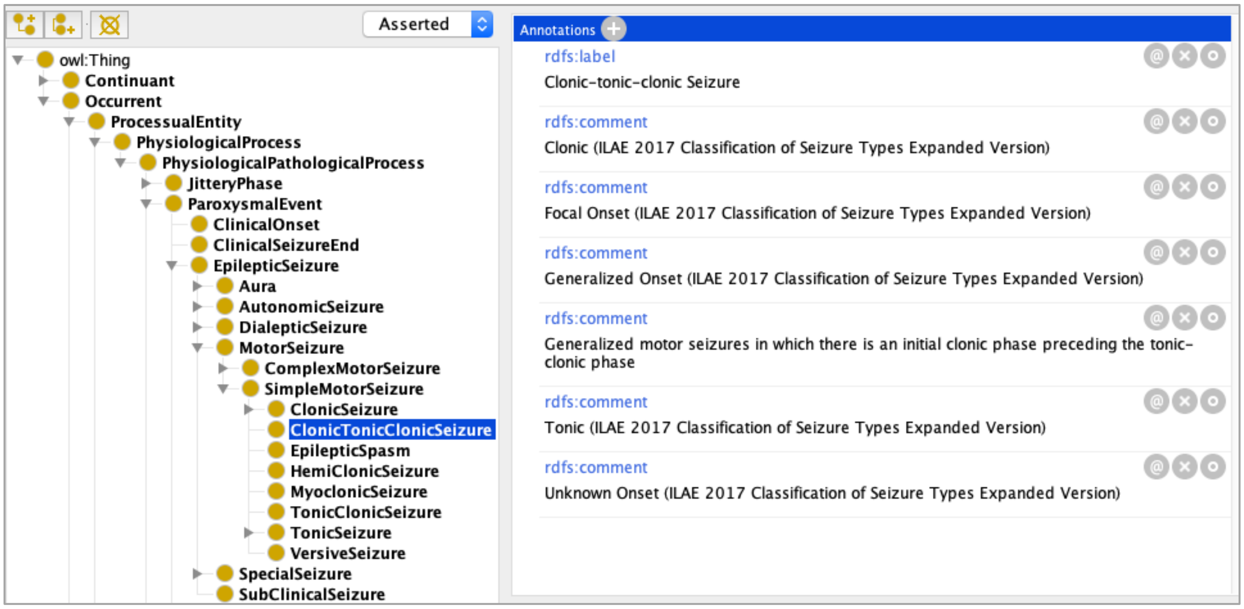


**Figure S2**: Multiple mappings created between ontology class and the ILAE 2017 classification of seizure types expanded version.

**References:**


1. Brickley D, Guha, R.V. RDF Schema 2004 [Available from: http://www.w3.org/TR/rdf-schema/.
2. Wu Z, Eadon, G., Das, S., Chong, E.I., Kolovski, V., Annamalai, M., Srinivasan, J., editor Implementing an inference engine for RDFS/OWL constructs and user-defined rules in oracle. IEEE 24th International Conference Data Engineering (ICDE); 2008: IEEE.
3. Hitzler P, Krötzsch, M., Parsia, B., Patel-Schneider, P.F., Rudolph, S. OWL 2 Web Ontology Language Primer. World Wide Web Consortium W3C; 2009.
4. Giannangelo K, Fenton, S. SNOMED CT Survey: An Assessment of Implementation in EMR/EHR Applications. Perspect Health Inf Manag 2008;5:7.
5. Doquire G, Verleysen, M. Feature selection for multi-label classification problems. . International work-conference on artificial neural networks: Springer Berlin Heidelberg; 2011. p. 9-16.
6. Pedregosa F, Varoquaux, G., Gramfort, A., Michel, V., Thirion, B., Grisel, O., Blondel, M., Prettenhofer, P., Weiss, R., Dubourg, V., Vanderplas, J. Scikit-learn: Machine learning in Python. Journal of machine Learning research. 2011;12:2825-30.
7. Breiman L. Random forests. Machine learning. 2001;45(1):5-32.
8. Olson D. L. DD. Advanced Data Mining Techniques2008.
9. Bouckaert RR, Frank, E. Evaluating the replicability of significance tests for comparing learning algorithms. Pacific-Asia conference on knowledge discovery and data mining: Springer, Berlin, Heidelberg; 2004. p. 3-12.
